# Potential Phytotherapy of DSS-Induced Colitis: Ameliorating Reactive Oxygen Species-Mediated Necroptosis and Gut Dysbiosis with a New *Crataegus pinnatifida* Bunge Variety—Daehong

**DOI:** 10.3390/antiox13030340

**Published:** 2024-03-12

**Authors:** Kang-In Lee, Yousang Jo, Heung Joo Yuk, Sun-Young Kim, Hyungjun Kim, Hye Jin Kim, Soo-Keol Hwang, Ki-Sun Park

**Affiliations:** 1 KM Science Research Division, Korea Institute of Oriental Medicine, Daejeon 34054, Republic of Korea; popigletoh@kiom.re.kr (K.-I.L.); joys@kiom.re.kr (Y.J.); yukhj@kiom.re.kr (H.J.Y.); heyjoon73@kiom.re.kr (H.K.); 2 College of Pharmacy, Chungbuk National University, Cheongju 28160, Republic of Korea; sun9412@sjtp.or.kr; 3 Department of Future Convergence Industry, Bio Health Industry Team, Sejong Technopark, Sejong-si 30141, Republic of Korea; 4 KM Convergence Research Division, Korea Institute of Oriental Medicine, Daejeon 34054, Republic of Korea; kimhyejin@kiom.re.kr; 5 Solwon Biotechnology, 3899, Sejong-ro, Jeonui-myeon, Sejong-si 30005, Republic of Korea

**Keywords:** inflammatory bowel disease, gut microbiota, *Crataegus pinnatifida* Bunge, Daehong, necroptosis, epicatechin

## Abstract

Developing new plant varieties plays a crucial role in competitiveness in the agricultural and food industries and enhancing food security. Daehong (DH) is a new variety of *Crataegus pinnatifida* Bunge (CP); however, its physiological functions and potential as a nutraceutical ingredient remain unknown. Here, the efficacy of DH on inflammatory bowel disease (IBD) was investigated using dextran sulfate sodium (DSS)-induced colitis mice, and its relative pharmacological effects were analyzed against CP. DH improved colitis-induced weight loss, colon shortening, and inflammatory responses and reduced intestinal permeability. The reactive oxygen species (ROS)-mediated necroptotic signal that triggers enterocyte cell death in DSS-induced colitis was effectively controlled by DH, attributed to epicatechin. DSS-induced gut dysbiosis was recovered into a healthy gut microbiome environment by DH, increasing beneficial bacteria, like *Akkermansia muciniphila*, and changing harmful bacteria, including *Bacteroides vulgatus* and Peptostreptococcaceae. DH shows potential as a dietary or pharmaceutical ingredient to promote gut health and to prevent and treat IBD.

## 1. Introduction

The development of new plant varieties is vital to address global challenges in agriculture, the environment, and food security. These new varieties offer the potential to increase yields, reduce environmental impact, enhance resilience to climate change, and promote healthier and more sustainable food systems [1,2].

Daehong (DH) has been developed as a new cultivar to enhance the utility of *Crataegus pinnatifida* Bunge (CP) as a food and pharmaceutical material. CP, commonly known as Chinese hawthorn or Shan Zha in traditional Chinese medicine, has a long history of use for its various biological effects and health benefits [3]. CP is rich in antioxidants, including flavonoids and polyphenols. These compounds help protect cells from oxidative damage and may play a role in reducing the risk of chronic diseases; furthermore, CP has been found to have a digestive-stimulating effect. It can help relieve indigestion, abdominal bloating, and gas, which is consistent with its traditional use [4]. CP helps with weight management by improving digestion and supporting metabolic processes, which can be beneficial for those looking to control their weight. Although CP exhibits outstanding pharmacological effects, its small fruits with large seeds pose limitations in terms of diverse culinary and medicinal applications. To address this gap, a new cultivar, DH, has been developed to increase the size of the hawthorn fruit, thus improving the yield of fleshy fruit (the morphological differences between CP and DH are explained in Supplementary Table S1). Furthermore, research is essential to compare and analyze the pharmacological efficacy of CP and DH, yet there is no known biological efficacy related to this.

Inflammatory bowel disease (IBD) is a group of chronic inflammatory conditions that primarily affect the gastrointestinal tract [5]. IBD was historically more prevalent in regions like North America and Europe, but there has been a notable increase in its occurrence worldwide, including Asia [6]. IBD mainly comprises Crohn’s disease, a chronic condition that can develop in any part of the digestive system, and ulcerative colitis, which specifically affects the lower colon. The precise etiology of IBD remains unclear, but potential causative factors may include a blend of genetic, environmental, and immunological components [7]. Of note, dysbiosis within the gut microbiome has recently emerged as a potential contributor to IBD [8]. Therefore, management of IBD necessitates a comprehensive therapeutic approach, encompassing pharmacotherapy aimed at mitigating inflammation and damping down aberrant immune responses. Anti-inflammatory drugs, immunosuppressants, and biologics are among the medications used to manage digestive tract symptoms. Nutritional therapy is an important part of symptom management and can promote overall health. In some cases, surgical intervention may be required to remove damaged sections of the digestive tract. Nevertheless, medications may have long-term side effects, including an increased risk of infections and certain cancers [9,10]. This has resulted in continued inquiry into alternative and complementary treatments, including changes in diet, use of probiotics, and herbal supplements as well as a focus on the development of safer and more targeted therapies [11,12,13].

In this study, we aimed to compare and analyze the efficacy of DH and CP in improving colitis using an IBD animal model as well as to investigate the active compounds of DH through biochemical analysis. In addition, we aimed to clarify the protective mechanisms of DH on enterocytes in the intestine and the crosstalk with gut microbiota. This comparative study examines the pharmacological effectiveness of DH and CP, which may reveal the potential benefits of the new variety and its suitability for food and health-related sectors. Therefore, this research could positively impact the food industry, health research, and agriculture.

## 2. Materials and Methods

### 2.1. Preparation of Sample and HPLC Analysis of CP and DH

**Figure 1:**
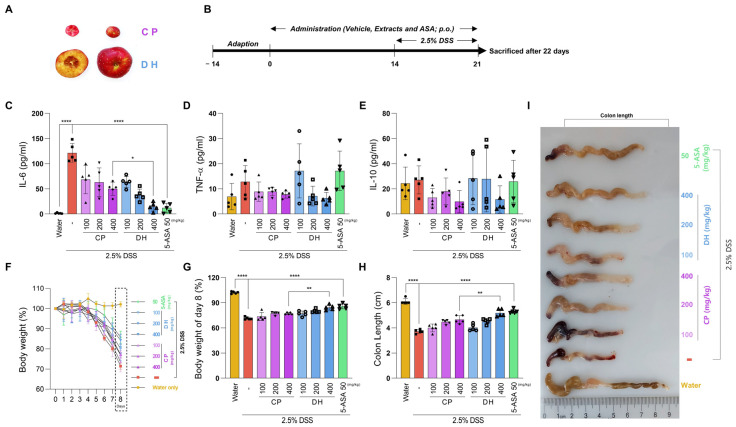
DH protects DSS-induced colitis. (**A**) Comparison of the appearance features of CP and DH. (**B**) Experimental design of oral administration to mice. (**C**–**E**) Serum cytokines of mice using ELISA. (**F**) Changes in body weight by date. (**G**) Weight changes on the eighth day after administration. (**H**,**I**) Colon length changes. n = 5 for each group. * *p*  <  0.05, ** *p*  <  0.01, and **** *p*  <  0.0001 (data were analyzed using ANOVA).

CP and DH fruits were supplied by Solwon Biotechnology (Sejong-si 30005, Republic of Korea). CP and DH were cultivated in the same area, located in Sejong-ro 3899, Sejong-si, Republic of Korea, and harvested on the same date. DH is a newly developed variety created through crossbreeding of different varieties of CP and has been mass produced through grafting. DH is currently registered as a unique Korean variety (No. 1010-10, Grant Number 81; Figure 1A). The breeding method of DH involves collecting large-fruited and thornless *Crataegus pinnatifida* trees from traditional *Crataegus pinnatifida* forests and performing artificial pollination. Subsequently, grafting methods with low mutation occurrence and rapid fruiting are utilized to secure the population. The fruits collected were freeze dried after seed removal and stored in a KIOM freezer at −20 °C until further analysis. Dried CP and DH were extracted with 70% ethanol under reflux for 6 h at 90 °C and then lyophilized after filtration. The freeze-dried extract was repeatedly sonicated for 30 min at 25 °C using methanol, and the supernatant was separated by centrifugation. For the quantitative HPLC analysis of standard components, epicatechin was obtained from Chemfaces (Cat# CFN98781, Wuhan, China). Individual components were simultaneously analyzed with extracts of CP and DH using an Agilent 1260 Infinity LC and an XBD-C18 column (4.6 mm × 50 mm; i.d., 5 µm) at a flow rate of 1 mL/min. The HPLC diode array detector measured at 280 nm, and chromatographic data were interpreted using the LabSolutions Multi-PDA software (https://www.shimadzu.eu/software-list, Shimadzu Corporation, Kyoto, Japan). The linear gradient was optimized using two mobile phases (A: water containing 0.2% TFA; B: ACN) as follows: 0 min, 10% B; 0–55 min, 25% B; 55–57 min, 0–100% B; 57–72 min, 100% B; 72–74 min, 100–10% B; and 74–84 min, stabilized to 10% B. All extraction and chromatographic solvents were of HPLC grade (J. T. Baker, Phillipsburg, NJ, USA).

### 2.2. Animal Study

To mimic chronic ulcerative colitis, an animal model was constructed under the following conditions; all animal experiments were conducted at the Korea Institute of Oriental Medicine (KIOM, Republic of Korea). Female C57BL/6 mice, aged six weeks and weighing 18 to 20 g, were purchased from Dooyeol Biotech in Seoul, Republic of Korea. They were accommodated under standard laboratory conditions, provided with *ad libitum* access to both water and food, and allowed a two-week acclimation period. Forty-five mice were randomly divided into the following groups (the cage never housed more than five mice at once): control (water only), 2.5% (*w*/*v*) DSS (MW 36 to 50 kDa; MP Biomedicals, Solon, OH, USA) only, DSS plus CP (100, 200, 400 mg/kg/day), DH (100, 200, 400 mg/kg/day), and 5-aminosalicylic acid (5-ASA; Cat# A3537, Sigma Aldrich, St. Louis, MO, USA; 50 mg/kg/day; n = 5/group). CP and DH were dissolved in distilled water, and 5-ASA was dissolved in 0.1 M HCl. CP, DH, and 5-ASA were orally administered once daily for 3 weeks. Over the final 7 days (from day 14 to day 21), mice were provided with drinking water containing 2.5% DSS ad libitum. The weights of the mice were recorded daily for the last 8 days. All mice were euthanized by intraperitoneal injection of tribromoethanol Avertin (300 mg/kg or 7.5 mg/mice, Cat. T48402, Sigma Aldrich, St. Louis, MO, USA), and the colon and blood were immediately harvested. All protocols used in this study are illustrated in Figure 1A. All protocols involving animals were performed in accordance with the Korea Institute of Oriental Medicine (KIOM-22-107) and the National Institutes of Health guide for the care and use of laboratory animals (NIH publication No. 8023, revised 1978).

### 2.3. Cell Culture

Caco2 and HT-29 cells were acquired from the American Type Culture Collection (ATCC, Manassas, VA, USA). Caco2 cells were incubated with Eagle’s Minimum Essential Medium (EMEM; Cat# HTB-37, ATCC, Manassas, VA, USA) containing 20% fetal bovine serum (FBS, Cat# 12662029, Thermo Scientific, Waltham, MA, USA), 100 U/mL penicillin, and 100 U/mL streptomycin (Cat# 10378016, Thermo Scientific). HT-29 cells were maintained in Dulbecco’s Modified Eagle’s Medium (DMEM; Cat# 11965092, Life Technologies, Waltham, MA, USA) containing 10% FBS (Cat# 12662029, Thermo Scientific), 100 U/mL penicillin, and 100 U/mL streptomycin (Cat# 10378016, Thermo Scientific). Human intestinal epithelial primary cells (InEpC) were obtained from Cell Biologics (Cat# H6047, Cell Biologics, Chicago, IL, USA) and cultured in a complete epithelial medium containing a supplement kit (Cat# H6621, Cell Biologics, IL, USA). The InEpC cells used in this study had a low passage number (<4 splitting cycles). The SZ complex, Smac mimetic (Cat# HY-12600, South Brunswick Township, NJ, USA), and z-VAD-fmk (Cat# HY-16658, South Brunswick Township) were purchased from MedChemExpress (Monmouth Junction, NJ, USA). The SZ complex was dissolved in dimethyl sulfoxide (DMSO).

### 2.4. Cell Viability Assay

We prepared 1 × 10^3^ cells in 96-well plates. All samples prepared in DMSO (DH, CP, and epicatechin) were cultured for 24 to 48 h, followed by treatment with an aqueous, non-radioactive cell proliferation solution (MTS) (Cat# G3582, Promega, Madison, WI, USA) for 30 min to 1 h at 37 °C. Absorbance was measured at 490 nm.

### 2.5. Immunohistochemistry (IHC)

Before conducting IHC, deparaffinization and hydration were conducted three times for 5 min each using xylene and ethanol in distilled water. Following that, the slides underwent heating in Antigen Retrieval Solution (Cat# ab93678, Abcam, Cambridge, MA, USA) for 20 min, followed by a 15-min incubation with 0.3% hydrogen peroxide. Slides prepared in distilled water were then treated with Sea-Block solution for 10 min (Cat# 37527, Abcam). The primary antibody (phosphorylated RIP3, Cat# 91702, Cell Signaling, Beverly, MA, USA) was diluted in DAKO solution (Cat# S080983-2, Agilent, Santa Clara, CA, USA). The slides were washed three times with phosphate buffered saline (PBS), and the secondary antibody was incubated for 1 h at 25 °C. The slides were exposed to the 3,3′-Diaminobenzidine solution to detect target genes and counterstained with hematoxylin to detect nuclear (Leica, Buffalo Grove, IL, USA). Terminal deoxynucleotidyl transferase-mediated dUTP nick-end labeling (TUNEL) staining was performed according to the manufacturer’s guidelines. Tissue samples on slides, after deparaffinization, underwent Proteinase K digestion at 55 °C for 30 min to 1 h, followed by staining using the TUNEL detection kit (Cat# ab206386, Abcam, Cambridge, MA, USA). Histological scoring was quantified using ImageJ version 1.52a software.

### 2.6. Cytokine Assay

For the quantification of IL-6, IL-10, and TNFα levels from the serum, enzyme-linked immunosorbent assay (ELISA) was performed (IL-6, Cat# ab222503; IL-10, Cat# ab100697; TNFα, Cat# 208348, Abcam, Cambridge, UK). The cytokine assay was conducted following the manufacturer’s guidelines, and the optical density was examined using an ELISA microplate reader (BioTek Synergy HTX; Agilent, Winooski, VT, USA).

### 2.7. Cell Permeability Assay

For the tight junction permeability analysis, Caco2 cells (0.7 × 10^5^ cells/well) were seeded on 1.12 cm^2^ polyethylene terephthalate Transwell filters in a 12-well plate with a 0.4-μm pore size (Cat# CLS3460, Corning, NY, USA). The cells were allowed to reach confluence, after which extracts and their components were added. After 24 h, the complete medium was replaced with a medium containing a lipopolysaccharide (LPS, 1 μg/mL) and interferon-gamma (IFNγ, 10 ng/mL) and incubated for 48 h. To measure transepithelial electrical resistance (TEER) values across cell tight junctions, Millicell^®^ ERS-2 (Electrical Resistance System) (Cat# MERS00002, Millipore, MA, USA) was used.

### 2.8. Levels of ROS Production

The levels of ROS production were assessed utilizing a flow cytometer along with 2′,7′-dichlorodihydrofluorescein diacetate (DCFDA) (Cat# D399, Invitrogen, MA, USA). A total of 1 × 10^6^ cells were plated in 6-well plates and pre-treated with each concentration of DH, CP, and epicatechin at 37 °C for 30 min. Then, the positive control SZ, which induces necroptosis, was exposed for 12 h. The cells were treated with 10 μmol/L DCFDA at 37 °C for 30 min, followed by subsequent procedures according to the manufacturer’s protocol. The quantitative analysis of fluorescent ROS was conducted using a flow cytometer (BD LSRFortessa X-20, Becton-Dickinson, San Jose, CA, USA). 

### 2.9. Annexin V-Propidium Iodide Assay

Necroptotic cell death was determined using an Annexin V-FITC Analysis Kit (Cat# BD556547, BD, NJ, USA). In brief, 1.2 × 10^6^ InEpC and HT-29 cells were seeded in 6-well plates and were pretreated with DH, CP, and epicatechin for 30 min and subsequently treated with SZ for 12 h to induce necroptotic cell death. Collected cells were mixed with 200 μL of a buffer containing 5 μL of an annexin V-FITC reagent for 30 min and then exposed with 5 μL of PI for 10 min at 25 °C in the dark room. The fluorescence intensities were measured using a flow cytometer (BD LSRFortessa X-20, Becton-Dickinson).

### 2.10. Immunoblotting

We seeded 1.2 × 10^6^ cells in 6-well plates, and cell lysates (30 to 50 µg) were obtained using mPER buffer (Cat# 78501, Thermo Scientific) supplemented with a protease inhibitor cocktail (Cat# 4693116001, Roche, Basel, Switzerland). All antibodies were diluted with a signal enhancer solution (HIKARI, Cat# 02267-41, NACALAI, Kyoto, Japan). Protein samples were loaded onto 4–20% TGX gels (Cat# 4561094, Bio-Rad) with molecular weight markers (Cat# 1610374, Bio-Rad, CA, USA). Electrophoresis was conducted at 100 volts for 100 min, and gels were transferred onto a polyvinylidene difluoride membrane (Cat# 1620177, Bio-Rad, CA, USA). Protein bands were detected using SuperSignal West Dura Extended Duration substrate (Cat# 34075, Thermo Scientific). The information for all antibodies is listed in Supplementary Table S2. To obtain digital images, the ChemiDoc MP imaging system (Bio-Rad, Hercules, CA, USA) was utilized.

### 2.11. 16S rRNA Gene Sequencing-Based Microbiome Profiling

Fecal microbiome profiles were measured using 16S rRNA gene sequencing. The FastDNA^®^ spin kit for soil (MP biomedicals, CA, USA) was used for DNA extraction. V3-V4 hypervariable regions of the 16S rRNA gene were amplified to measure the bacterial composition. The 341F (forward: 5′-TCGTCGGCAGCGTCAGATGTGTATAAGAGACAGCCTACGGGNGGCWGCAG-3′) and 805R (reverse: 5′-GTCTCGTGGGCTCGGAGATGTGTATAAGAGACAGGACTACHVGGGTATCTAATCC-3′) primers were used for amplicon PCR to target the V3-V4 regions, and index PCR was performed on the amplicons. The PCR products were analyzed by paired end sequencing using the Illumina MiSeq platform (Illumina, CA, USA). The 16S sequence data were clustered using USEARCH [14], and the operational taxonomy unit (OTU) profiles were identified by assigning to the PKSSU 4.0 database [15]. The preceding analyses were performed by CJ Bioscience, Inc. (Seoul, Republic of Korea)

### 2.12. Differential Abundance Analysis for Microbiome Profiles

A differential abundance analysis was performed to identify differentially abundant OTUs for each group. ALDEx2 [16] and ANCOM-BC2 [17] were used due to their consistency in the comparative study [18]. The statistical significance (*p*-value) of each OTU was evaluated by a generalized linear model in ALDEx2 and ANCOM-BC2 separately. OTUs with a Benjamini–Hochberg adjusted *p* < 0.05 from either of the two methods were selected as differential OTUs. OTUs differentially abundant in both the control vs. DH and DSS vs. DSS + DH were selected to find OTUs, which compensated for the alteration from DSS.

### 2.13. Metagenome Analysis

Amplicon sequence variants (ASVs) of 16S microbiome data were identified by DADA2, and the metagenome profile was reconstructed from ASVs using PICRUSt2 software (Version 2.5.2) [19,20]. The metagenome profile was merged into the pathway activity profile, which was annotated to the MetaCyc database [21]. Differentially abundant pathways were identified by ALDEx2; pathways with a Benjamini–Hochberg adjusted *p* < 0.05 in both the control vs. DH and DSS vs. DSS + DH were selected.

### 2.14. Statistical Analysis

All statistical analyses for in vivo and in vitro experiments were performed with GraphPad Prism software (Version 9.0) and one-way analysis of variance (ANOVA) (Prism, San Diego, CA, USA). Data are expressed as the mean ± standard error (SE), and significance levels were established at * *p* < 0.05, ** *p* < 0.01, *** *p* < 0.001, and **** *p*  <  0.0001.

All microbiome analyses were performed with the mia package in R software (Version 4.3.2). The number of observed species, Shannon’s diversity, and Faith’s phylogenetic diversity were measured for alpha diversity, and Bray–Curtis dissimilarity was used as beta diversity for principal coordinate analysis (PCoA). The statistical significance of the alpha diversity was evaluated by Wilcoxon’s rank-sum test. All *p*-values in differential abundance analysis were adjusted by the Benjamini–Hochberg procedure.

## 3. Results

### 3.1. DH Is More Effective than CP in Alleviating DSS-Induced Colitis

The new variety DH is approximately five times heavier than CP and has a diameter three times larger (Figure 1A and Table S1). While the biological effects of DH are largely unknown, CP is well known for its traditional anti-colitis and anti-inflammatory effects [13]. Therefore, the DSS-induced colitis model was suitable for the comparative analysis of the biological potency of DH and CP. DH and CP were administered orally for 21 days, with 2.5% DSS for the last 7 days (Figure 1B). Then, 5-ASA was used as a positive control to improve colitis [22]. First, IL-6, TNFα, and IL-10 were analyzed to investigate the anti-inflammatory effects of DSS. Although DSS drastically increased IL-6, both CP and DH effectively suppressed IL-6 production at low concentrations (Figure 1C). Interestingly, the anti-inflammatory properties of DH were improved over CP, showing a significant difference at a concentration of 400 mg/kg. Although not statistically significant, increased pro-inflammatory cytokine TNFα by DSS showed a tendency to decrease by DH compared to CP (Figure 1D). However, no significant changes were observed for IL-10, an anti-inflammatory cytokine (Figure 1E). Then, the potential of DH to alleviate colitis was confirmed through its anti-inflammatory effects. DSS was administered for 7 days, and body weight was recorded until the 8th day. Both groups administered with CP and DH showed dose-dependent recovery of body weight, with a significant difference observed in the DH group compared to the CP group on the 8th day (Figure 1F,G). Furthermore, the length of the colon shortened by DSS also improved by approximately 10% in the group administered DH compared to the group administered CP (Figure 1H,I). These results are consistent with the previous inhibitory effects of IL-6 and TNFα. Therefore, we suggest that the anti-inflammatory and alleviating effects of DH colitis are superior to those of CP, further suggesting that it could be a potential natural product for the targeting of disease.

### 3.2. DH Improves the Paracellular Permeability of Enterocytes Impaired by LPS and INFγ

**Figure 2:**
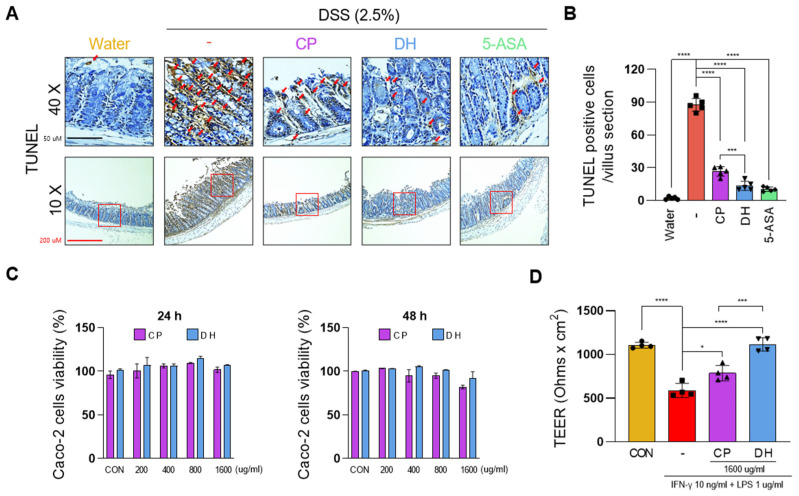
DH recovers DSS-induced damage in the intestinal epithelium. (**A**) TUNEL staining in colon tissue. Red arrows indicate TUNEL-positive cells. (**B**) TUNEL positive cells from colon tissue. (**C**) Cell viability test by CP and DH. (**D**) Effects of the culture medium on TEER; n = 5 for each group. * *p*  <  0.05, *** *p*  <  0.001, and **** *p*  <  0.0001 (data were analyzed using ANOVA).

To investigate the correlation between changes in colon length by DSS and enterocyte death, the TUNEL assay was performed at a concentration of 400 mg/kg, which showed the most significant change. As we hypothesized, the DSS-administrated group could observe death in most enterocytes. In the group treated with CP, DH, and 5-ASA, enterocyte death was suppressed, but, consistent with previous results, DH improved enterocyte death by approximately 50% more than CP (Figure 2A,B).

Damaged enterocytes contribute to intestinal leakage; therefore, protecting them is crucial to inhibiting IBD [23]. To compare the effects of suppressing cell permeability, differentiated Caco2 cells were treated with LPS and INFγ to mimic the intestinal permeation environment. DH and CP did not exhibit cytotoxicity up to a concentration of 1600 mg/mL (Figure 2C), and were pre-treated for 24 h. Subsequently, they were co-cultured with LPS and INFγ for 5 days to measure the TEER value (Figure 2D). Interestingly, both DH and CP suppressed cell permeability, but DH was approximately 20% more effective in inhibiting cell permeability compared to CP. Thus, we demonstrate that DH suppresses intestinal leakage by protecting enterocytes.

### 3.3. DH Protects Enterocyte Death by Suppressing Necroptosis of Immune Response in DSS-Induced Colitis

**Figure 3:**
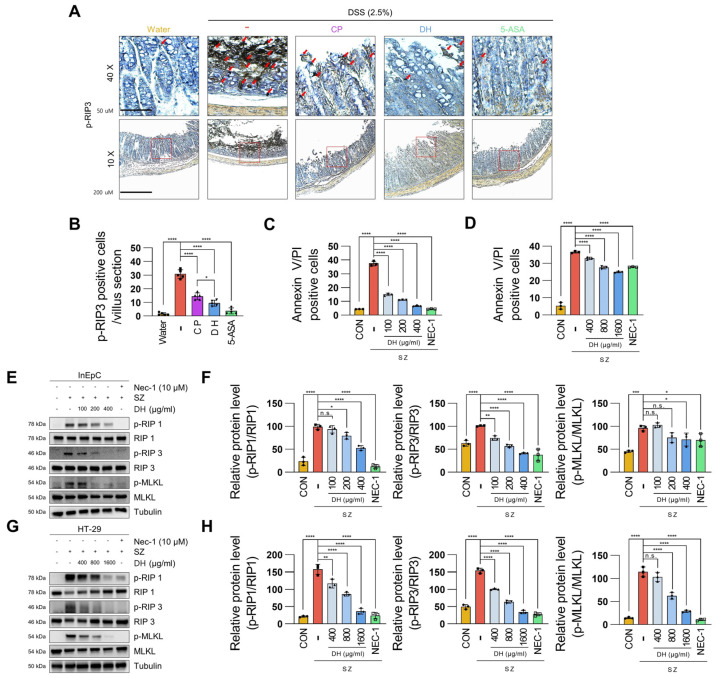
DH regulates necroptosis in enterocytes. (**A**,**B**) p-RIP3, necroptosis marker, expression levels in colon tissues; n = 5 for each group. Red arrows indicate p-RIP3 positive cells. (**C**) The values represent the percentage of InEpC cells by FACS analysis (PI+ annexin V+; necroptosis); n = 3 for each group. (**D**) The values represent the percentage of HT-29 cells by FACS analysis (PI+ annexin V+; necroptosis); n = 3 for each group. (**E**,**F**) Protein levels of the necroptosis marker in InEpC cells; n = 3 for each group. (**G**,**H**) Protein levels of the necroptosis marker in HT-29 cells; n = 3 for each group. * *p*  <  0.05, ** *p*  <  0.01, *** *p*  <  0.001, and **** *p*  <  0.0001 (data were analyzed using ANOVA); n.s., not significant.

Enterocyte death by necroptosis is a pivotal intracellular signal that causes colitis along with intestinal leakage [24]. To investigate whether DH can modulate necroptotic signals, we examined the expression of the necroptosis marker p-RIP3 [25]. Although necroptosis significantly decreased in both groups treated with CP and DH, DH exhibited superior effectiveness in suppressing necroptosis compared to CP in colitis tissues (Figure 3A,B). Therefore, it is essential to study the mechanism by which DH regulates necroptosis in enterocytes. To address this question, after induction of necroptosis from InEpC and HT-29 cells to Smac mimetic with z-VAD-fmk (SZ) [26], we verified the anti-necroptotic effect by FACS analysis. In both cells, the increase in necroptotic cell death was decreased by DH in a dose-dependent manner (Figure 3C,D, Supplementary Figure S1). Furthermore, DH significantly decreased the protein levels of phosphorylated RIP1, RIP3, and MLKL, the necroptosis markers activated by SZ (Figure 3E–H), consistent with the FACS analysis. These results suggest that DH protects enterocyte death through a mechanism that regulates necroptotic signaling.

### 3.4. DH Regulates SZ-Induced ROS Production via the JNK/p38-MAPK/COX2 Pathway

**Figure 4:**
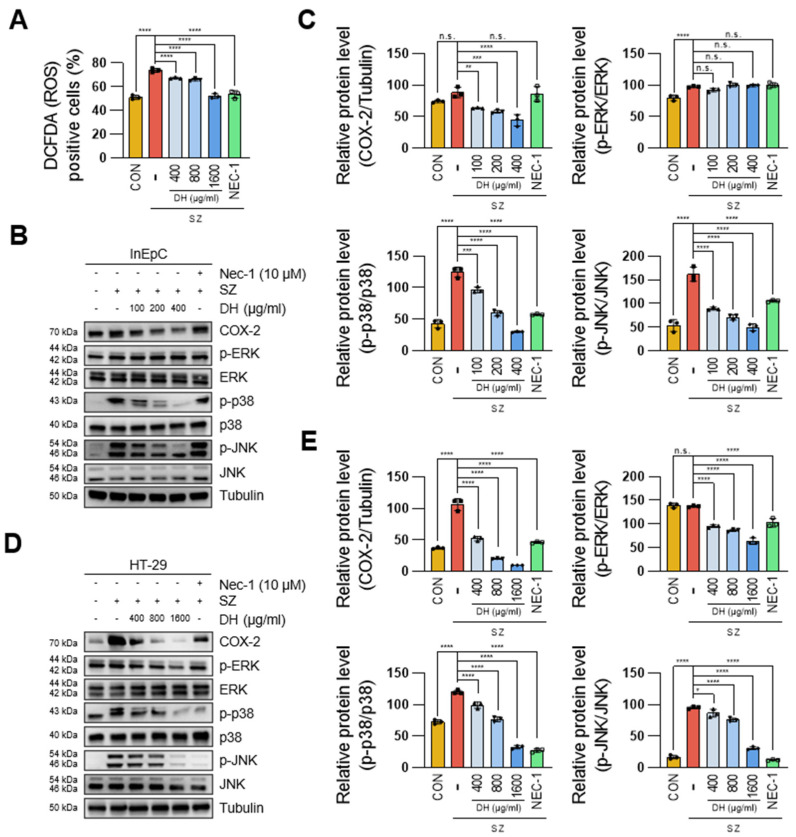
DH inhibits ROS production and related genes in SZ-induced necroptosis. (**A**) Number of positive cells for DCFDA fluorescence in Supplementary Figure S2. (**B**,**C**) Protein levels of ROS production-related genes in InEpC cells. (**D**,**E**) Protein levels of ROS production-related genes in HT-29 cells. * *p*  <  0.05, ** *p*  <  0.01, *** *p*  <  0.001, and **** *p*  <  0.0001 (data were analyzed using ANOVA); n.s., not significant.

ROS are not only known triggers to promote necroptosis but can also exacerbate colitis [26]. To investigate whether DH regulates necroptosis via ROS signaling, we first examined the levels of ROS production through FACS analysis. SZ-induced necroptosis resulted in approximately a 25% increase in DCFDA-positive cells, whereas DH dose-dependently decreased ROS production (Figure 4A, Supplementary Figure S2). Furthermore, DH dose dependently decreased the levels of phosphorylated p38-MAPK and JNK, ROS-related signals activated by SZ, while also suppressing the expression of COX2, an ROS target gene (Figure 4B–E). These results emphasize that DH can regulate enterocyte necroptosis by suppressing ROS production.

### 3.5. Epicatechin Is the Bioactive Component of DH Responsible for the Regulation of Necroptosis

**Figure 5:**
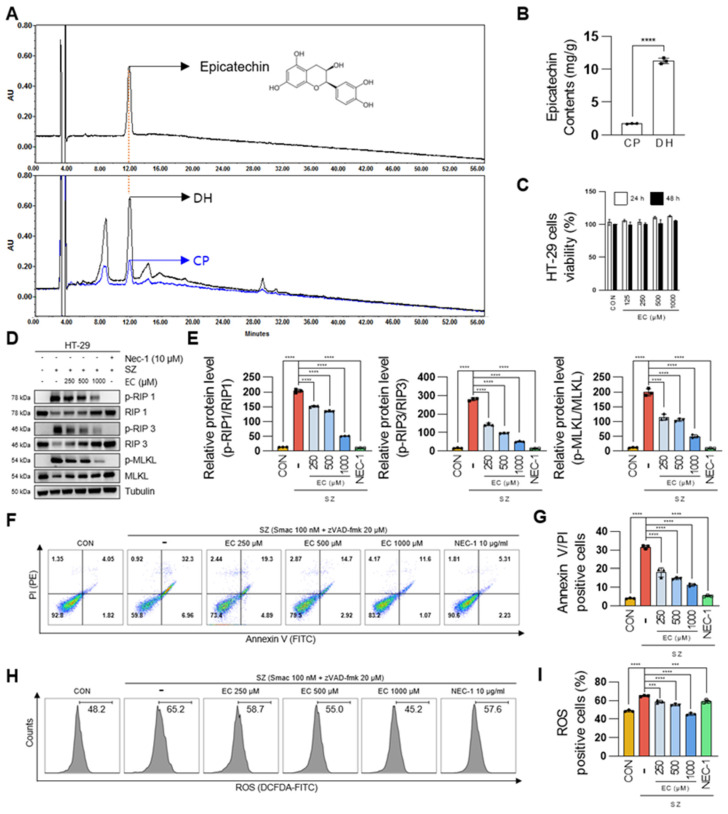
The epicatechin component of DH regulates necroptosis in enterocytes. (**A**,**B**) Comparison of epicatechin content in DH and CP using HPLC. (**C**) Epicatechin cell viability test. (**D**,**E**) Protein levels of the necroptosis marker by epicatechin. (**F**) FACS analysis of annexin V/PI-stained cells in HT-29 cells; n = 3 for each group. (**G**) Values represent the percentage of cells in F (PI+ annexin V+; necroptosis); n = 3 for each group. (**H**) Intracellular ROS levels by flow DCFDA fluorescence. (**I**) Number of positive cells for DCFDA fluorescence in H; n = 3 for each group. *** *p*  <  0.001 and **** *p*  <  0.0001 (data were analyzed using ANOVA); ns, not significant.

To analyze the components of DH and pharmacologically explain its therapeutic effect on colitis, we conducted an HPLC analysis and compared its composition to that of CP. Significantly different contents of various components were observed in DH (Figure 5A). The contents of epicatechin in DH and CP were analyzed using an HPLC at 280 nm, the maximum UV wavelength of epicatechin as the main component. The standard curve demonstrated a highly validated correlation coefficient (r^2^ > 0.999) and was linear and reproducible. Quantitative analysis confirmed that the epicatechin content in the extract with 70% EtOH was 11.3 mg/g in DH and 1.7 mg/g in CP per dry sample, respectively (Figure 5B). To investigate whether epicatechin regulates necroptosis in cellular systems, cytotoxicity studies were conducted, maintaining concentrations up to 1 mM for 48 h (Figure 5C). Pre-treatment with epicatechin for 30 min significantly suppressed the levels of phosphorylated RIP1/RIP3/MLKL activated by SZ in a dose-dependent manner (Figure 5D,E). Furthermore, epicatechin inhibited SZ-induced enterocyte cell death (Figure 5F,G) and significantly regulated ROS production (Figure 5H,I). Nec-1 was used as a positive control to inhibit necroptosis. In conclusion, we demonstrate that epicatechin, as a bioactive compound of DH, regulates necroptosis. 

### 3.6. DH Strengthens the Composition of the Fecal Microbiota in DSS-Induced Mice

**Figure 6:**
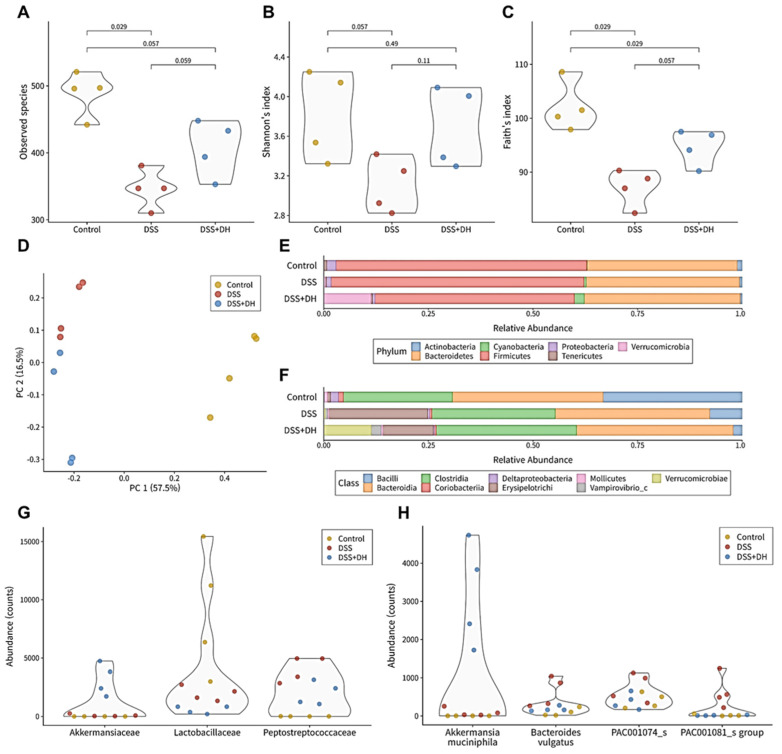
Comprehensive profile of the fecal microbiome. (**A**–**C**) Alpha diversity profiles of all samples evaluated by the number of observed species, Shannon’s index, and Faith’s phylogenetic index. (**D**) Principal coordinate analysis (PCoA) plots of the fecal microbiome based on Bray–Curtis dissimilarity. (**E**,**F**) Taxonomic compositions of the fecal microbiome at the phylum and class level. (**G**,**H**) Differentially abundant taxa at the family species level.

The gut microbiome plays a crucial role in the pathology of numerous intestinal diseases, including IBD [27]. Thus, we analyzed the fecal microbiome profile to discover microbial alterations induced by DSS and DH treatment. Alpha diversity indices served as comprehensive indicators of the microbial community in Figure 6A–C. DSS significantly reduced all three diversities compared to the control group. However, DH treatment of DSS mice significantly increased observed species and Faith’s index and restored Shannon’s index to the control group’s level. This suggests that DH strengthens the gut microbiome that DSS disrupts. Beta diversity was assessed to determine whether the microbial composition of the three experimental groups could be differentiated. PCoA was conducted using Bray–Curtis dissimilarity. The PCoA plots distinctly separated the three clusters, demonstrating that DSS-induced colitis and DH treatment alter the gut microbiome (Figure 6D).

At the phylum level, Firmicutes and Bacteroidetes constituted the majority (80–90%) of the fecal microbiome in all groups, similar to the normal gut microbiome (Figure 6E). However, Verrucomicrobia was significantly abundant only in the DSS + DH group. The class-level composition displayed more differences among the three groups (Figure 6F). The abundance of Bacilli, which comprises the well-known beneficial bacteria *Lactobacillus*, was reduced in the two DSS-administered groups, DSS and DSS + DH. In contrast, Erysipelotrichi was significantly more abundant in the DSS and DSS + DH group, as reported in inflammatory mouse models [28]. Nevertheless, the abundance of Erysipelotrichi in the DSS + DH group was about half of that in the DSS group, which implies that DH treatment could mitigate the DSS-induced alteration. Furthermore, the abundance of Verrucomicrobiae was significantly higher in the DSS + DH group, reflecting the observation at the phylum level.

### 3.7. DH Supports Beneficial Bacteria and Suppresses Harmful Bacteria in the Fecal Microbiota

Differential abundance analysis (DAA) was performed to discern the distinctive microbial signatures of each group using ALDEx2 and ANCOM-BC2. OTUs that are differentially abundant in both the control vs. DSS and DSS + DH vs. DSS were selected as microbial signatures that are altered by DSS but ameliorated by DH. Furthermore, we listed the IBD-related functionality of significant OTUs based on evidence from the literature (Supplementary Table S3).

At the family level, Akkermansiaceae, Lactobacillaceae, and Peptostreptococcaceae exhibited significant differences across the groups (Figure 6G). Akkermansiaceae was exclusively abundant in the DSS + DH group, indicating the DH-specific increase of the beneficial bacteria *Akkermansia muciniphila*. Lactobacillaceae decreased in both the DSS and DSS + DH groups. This suggests that DSS-induced colitis suppresses Lactobacillaceae, and DH-mediated amelioration is independent of Lactobacillaceae. Furthermore, Peptostreptococcaceae increased significantly in the DSS group and showed a reduction of approximately 60% (log2FC = 1.2) in the DSS + DH group. Previous studies have reported a positive association between Peptostreptococcaceae and IBD via parts of the host pathway, MAPK3 and VIPR1 [29]. Thus, inhibiting Peptostreptococcaceae could contribute to DH’s protective effect. At the genus level, *Akkermansia*, PAC001074_g, and PAC000186_g showed differential abundances. The alterations observed in *Akkermansia* and PAC001074_g can be attributed to changes at the single species level, as detailed in the subsequent section. However, PAC000186_g has a few reports regarding reproductive organs but not IBD.

As illustrated in Figure 6H, the *Akkermansia muciniphila*, *Bacteroides vulgatus*, PAC001074_s, and PAC001081_s groups showed differential abundances across the groups. *Akkermansia muciniphila* is substantially rich in the DSS + DH group and elucidates the DH-specific richness of its high-rank taxa, Verrucomicrobia, Akkermansiaceae, and *Akkermansia*. Furthermore, previous studies suggested that *Akkermansia muciniphila* provides numerous beneficial effects on intestinal health, including anti-inflammation effects, support of the gut barrier, and intestinal cell proliferation [30,31,32,33]. Therefore, the prebiotic effect of DH on *Akkermansia muciniphila* may constitute a central component of DH-mediated support for intestinal health. In contrast, *Bacteroides vulgatus* was significantly abundant in the DSS group, whereas it decreased close to the control group in the DSS + DH group. *Bacteroides vulgatus* is a well-known harmful bacteria that induces gut barrier dysfunction and ulcerative colitis [34]. Consequently, DH may contribute to the mitigation of inflammation by suppressing *Bacteroides vulgatus*. The PAC001074_s and PAC001081_s groups were also abundant in the DSS group and decreased in the DSS + DH group. There is limited information about these two species, but the PAC001081_s group is positively correlated with lipopolysaccharide (LPS)-induced systemic inflammation [35] and may be associated with the protective effect of DH. Therefore, DH could mitigate inflammation and support intestinal health through the support of beneficial bacteria such as *Akkermansia muciniphila* and the suppression of harmful bacteria, including Peptostreptococcaceae, *Bacteroides vulgatus*, and the PAC001081_s group.

### 3.8. DH Restores the Functionality of the Gut Microbiota to Improve Gut Health and Reduce Oxidative Stress

**Figure 7:**
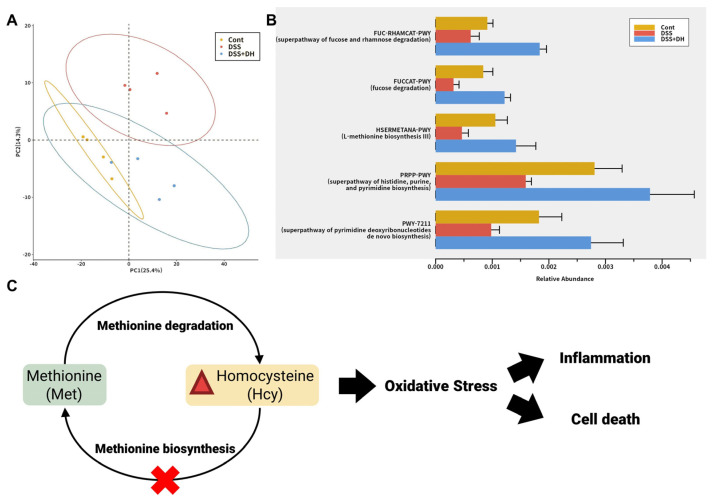
Functional composition of the fecal microbiome based on metagenome analysis. (**A**) Principal component analysis (PCA) plot of the pathway activity profile of the fecal microbiome. (**B**) Differentially abundant pathway activities among control, DSS, and DSS + DH groups. (**C**) Homocysteine-methionine cycle and its impact on gut health.

Metagenome analysis of the fecal microbiota was conducted to discover the impact of DH on the functional composition of the microbiota. The pathway activity profile was inferred based on the fecal microbiome profile. Like microbial composition, the DSS + DH group was distinguished from the DSS group and similar to the healthy control group (Figure 7A). Furthermore, a differential analysis was performed to find pathways that were weakened by DSS-induced colitis and restored by DH treatment, and fucose degradation and its superpathway, nucleotide synthesis, and methionine synthesis were selected (Figure 7B).

Restored pathways by DH illustrate how DH ameliorates DSS-induced colitis through gut microbiota. Fucose metabolism was reported to promote the production of propionate by *Akkermansia muciniphila*, a strain substantially increased by DH treatment, and propionate reduces intestinal inflammation and repairs the gut barrier [32,36]. Nucleotide synthesis is decreased in the microbiota in ulcerative colitis and Crohn’s disease, and previous studies suggested it may reflect the increase of auxotrophic pathobionts, which depend on nucleotide remnants of dead cells [37]. Methionine synthesis is an essential component of the homocysteine-methionine cycle and reduces homocysteine, which induces oxidative stress and inflammation [38,39]. The weakening of this pathway increases free homocysteine and may lead to ROS-mediated cell death and inflammation (Figure 7C). It is supported by clinical reports that hyperhomocysteinemia is common in IBD patients [40]. Considering the functions of these pathways, the restoration of the functionality of gut microbiota by DH results in the improvement of gut health and the reduction of oxidative stress.

## 4. Discussion

The ongoing development of new crop varieties is crucial for advancing agriculture and adapting to changing climate conditions. CP typically demonstrates excellent efficacy for gastrointestinal disorders and anti-inflammatory purposes. However, its functional ingredient capacity is hampered by its small plant size and large seeds. To overcome this limitation, a new variety called DH has been developed, but there have been no studies comparing its pharmacological efficacy with that of the existing CP. Therefore, the objective of this study was to explore the physiological advantages of DH and its potential as a dietary pharmacological ingredient for improving gut health. To achieve this, a DSS-induced chronic colitis animal model was used.

DH effectively suppressed the levels of IL-6 induced in response to colitis compared to CP. DH showed nearly identical potency to the positive control 5-ASA in improving colitis, especially at high concentrations. Despite not witnessing significant alterations in TNF-α and IL-10 levels in the current DSS-induced colitis model, we expect such modifications to occur with concentration and duration adjustments. DSS-induced colitis results in loss of body weight and shortening of the colon length, both of which were consistently improved by DH. DH, especially at a high concentration, demonstrated approximately 15% to 20% greater pharmacological efficacy compared to CP. These findings further demonstrate that DH offers superior protection to intestinal cells compared to CP, as indicated by measurements of changes in resistance values of colon tight junctions induced by INF-γ and LPS treatment, which can cause leakage of intestinal tissue.

Pierdomenico et al. [41] confirmed the activation of necroptotic signals in inflammatory tissue in 30 patients with ulcerative colitis and 33 patients with Crohn’s disease. While our findings do not definitively establish the induction of necroptosis in all cases of IBD, our research experience and the reported findings suggest that investigating target molecules that regulate necroptosis is a crucial avenue of research in combating IBD. These research results underscore the importance of studying necroptosis mechanisms in IBD for the discovery of diagnostic markers in molecular biology, identification of therapeutic targets, and drug development. In our study, we also identified the activation of necroptotic signals in the DSS-treated group in addition to severe cell death. Although it is a new discovery that CP, together with DH, decreases necroptotic signals, the superior effectiveness of DH at the same concentration highlights its greater potential as a dietary pharmaceutical substance. However, further research is needed to support the scientific efficacy of DH by analyzing the regulatory mechanisms of target genes in necroptosis-induced IBD. To clarify the pharmacological effects of DH, it is essential to perform a compositional analysis through chemical studies. HPLC analysis revealed that the concentration of epicatechin in DH is approximately eight times greater than in CP. Epicatechin is well known for its antioxidative and anti-inflammatory properties, and recent research has confirmed the improvement of colitis in animal models treated with epicatechin. These findings suggest that the colitis-improving effect of DH is significantly attributed to its high epicatechin content. DH and CP exhibit differences in the content of numerous components (Figure 5A). We are still identifying and characterizing the components of DH through mass and NMR analyses to expand our understanding of the pharmacological effects and mechanisms of DH.

Studies on the correlation between IBD and microbial communities are crucial to understand the onset and progression of IBD [42,43]. Furthermore, such studies have the potential to confirm the development of microbiome-based treatments for IBD. These treatments may involve strategies such as probiotics, prebiotics, or dietary interventions to restore a healthier gut microbial balance. In this study, the significant increase in the *Akkermansia muciniphila* strain in the gut resulting from DH consumption suggests the potential of DH to develop into a prebiotic. The increased presence of *Akkermansia muciniphila* is suggested to reduce the risk of metabolic diseases such as obesity and diabetes, as indicated by previous research, contributing to maintaining a healthy body weight through appetite regulation and improved insulin sensitivity [33]. *Akkermansia muciniphila* supports a healthy gut barrier by preventing the absorption of harmful pathogens and toxins, thus strengthening the intestinal mucosal layer [31,44]. In contrast, the *Bacteroides vulgatus* strain, which was decreased by DH consumption, is classified as a harmful microbe that can exacerbate gut inflammation [34]. However, more research is needed to establish a direct correlation between Bacteroides vulgatus and IBD. Furthermore, metagenomics analyses in this study revealed that DH-mediated modulation of gut microbiota can reduce homocysteine, a major source of oxidative stress (Figure 7C). This finding suggests the possibility of DH as an indirect antioxidant and a cell death inhibitor. These research findings suggest promising pharmacological effects of DH. The limitations of this study include the use of 16S rRNA gene-based intergroup comparative analysis, which provides insights into group-level comparisons but may not fully elucidate strain-specific microbiome profiles and behaviors. Therefore, further exploration of the organic relationship between DH and the gut microbiota will be necessary through a higher resolution analysis based on metagenomic sequencing. Furthermore, a substantial portion of the effects of the microbiome on gut health are mediated by the host’s response to microbial products, including peptides, saccharides, and bacterial extracellular vehicles (EVs). These host–microbiome interactions induced by DH can be clarified with the integration of microbiome profiling and gut-specific multiomics analyses.

## 5. Conclusions

**Figure 8:**
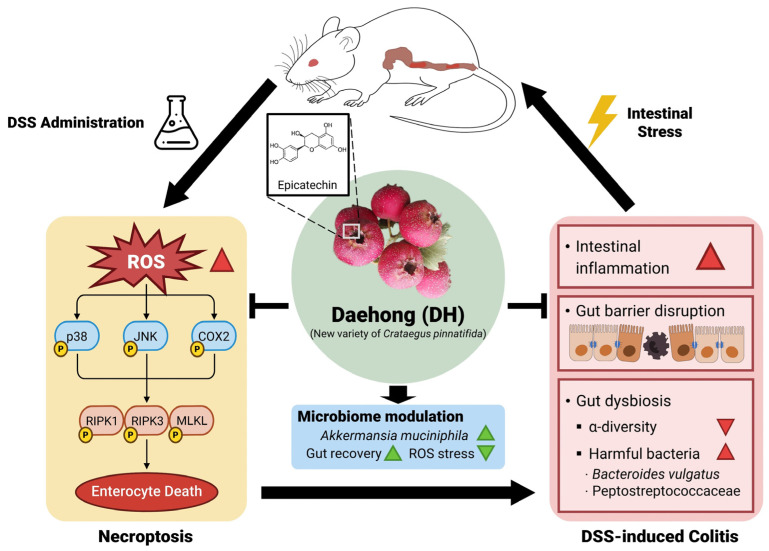
The proposed mechanism of DH in DSS-induced colitis mice model.

This study focuses primarily on investigating the potential of the new DH variety as a dietary material for gut health. DH improved colitis responses, increased body weight loss, and reduced colon length and tight junction collapse in a colitis animal model, showing superior efficacy compared to CP. To improve IBD, inhibiting the activated necroptosis mechanism is crucial, and DH could effectively control these necroptotic signals. These findings have led to the exploration of changes in the gut microbiome environment, ultimately contributing to the restoration of gut homeostasis in the colitis animal model by DH (Figure 8). New discoveries emphasize the need for variety development and highlight DH’s potential for IBD treatment and prevention.

## Data Availability

Data are contained within the article or Supplementary Materials.

## Supplementary Materials

The following supporting information can be downloaded at: https://www.mdpi.com/article/10.3390/antiox13030340/s1, Table S1. The morphological characteristics of DH and CP; Table S2. Antibodies for immunoblotting; Table S3. Literature evidences for the functionality of differentially abundant taxa; Table S4. The abbreviations used in this study; Figure S1. DH regulates necroptotic cell death; Figure S2. DH regulates ROS production.
