# Supplementary material for: Potential Phytotherapy of DSS-Induced Colitis: Ameliorating Reactive Oxygen Species-Mediated Necroptosis and Gut Dysbiosis with a New Crataegus pinnatifida Bunge Variety—Daehong
Source: Antioxidants (Basel). 2024 Mar 12;13(3):340. doi: 10.3390/antiox13030340 (PMC10968080; doi:10.3390/antiox13030340)
Supplement: Supplementary file 1 [file antioxidants-13-00340-s001.zip › antioxidants-2888107-supplementary.pdf]

Supporting Information for

Potential Phytotherapy of DSS-Induced Colitis: Ameliorating ROS-mediated Necroptosis and Gut Dysbiosis with a New *Crataegus pinnatifida* Bunge Variety—Daehong

Kang-In Lee[1,6], Yousang Jo[1,6], Heung-Joo Yuk[1], Sun-Young Kim[2,3], Hyungjun Kim[1], Hye Jin Kim[4], Soo-Keol Hwang[5,*] and Ki-Sun Park[1,*]

[1]KM Science Research Division, Korea Institute of Oriental Medicine, Daejeon 34054, Republic of Korea.

[2]College of Pharmacy, Chungbuk National University, Cheongju, 28160, Republic of Korea

[3]Department of Future Convergence Industry, Bio Health Industry Team, Sejong Technopark, Sejong-si 30141, Republic of Korea

[4]KM Convergence Research Division, Korea Institute of Oriental Medicine, Daejeon 34054, Republic of Korea.

[5]Solwon Biotechnology, Sejong-si 30005, Republic of Korea

[6]These authors contributed equally.

[*]Corresponding authors

E-mail address: sookeol@hanmail.net (S-K. H.), kisunpark@kiom.re.kr (K-S. P.)

Tel.: +82-42-868-9662

Supplementary Table S1. The morphological characteristics of DH and CP

Plant Characteristics	DH	CP
Fruit Weight	20~30g	3~5g
Fruit Diameter	2.5~4cm	1.5cm
Presence of Thorns	None	Present
Fruit Shape	Spherical, pentagonal stripes	Spherical
Number of Flowers per Cluster	12 on average	5~6, maximum 15
Flower Size	Large (approx. 2x of CP)	Small
Leaf Size	Approx. 10cm	Approx. 4cm
Leaf Base	Thin and long	Thick and short
Stem Color	Brown with white spots	Grey with white spots
Fresh Consumption	Available due to rich flesh	Unavailable, small and insipid
Fruit Skin Color	Large, clean, and non-bursting	Small and prone to blemishes
Fruit Color	Deep pink	Red or dark red
Storage Life	Long	Short
Leaf Underside	Clean	Prominent leaf veins with fine hairs
Fruiting Period	Early fruiting from the first year	Fruiting after 5 years
Propagation Method	Grafting (budding, veneer graft)	Seed Sowing
Growth Habit	Umbrella shape	Umbrella shape

Supplementary Table S2. Antibodies for immunoblotting

Antibody	Source	Catalog No.
RIP1	Cell signaling	#3493
p-RIP1	Cell signaling	#65746
RIP3	Cell signaling	#10188
p-RIP3	Cell signaling	#93654
MLKL	Cell signaling	#14993
p-MLKL	Cell signaling	#91689
p38	Cell signaling	#9212
p-p38	Cell signaling	#9211
ERK	Cell signaling	#9102
p-ERK	Cell signaling	#4370
JNK	Cell signaling	#3708
p-JNK	Cell signaling	#4668
COX-2	Abcam	Ab52237
Tubulin	Abcam	Ab7291

Supplementary Table S3. Literature evidences for the functionality of differentially abundant taxa. q-value refers to the p-value from ALDEx2 adjusted by Benjamini-Hochberg procedure.

**Taxon**	**DH-induced alteration (vs. DSS)**	**Gut-related functionality**
Peptostreptococcaceae	Decreased (q = 0.0421)	Harmful;<br>- Positively correlated with genes in IBD-related pathway, MAPK3, VIPR1, PYGB, NCK2.<br>- Negatively correlated with gut-protective gene, ANXA1.
Akkermansia muciniphila	Increased (q = 0.0092)	Beneficial;<br>- Reduced inflammation by the stimulation of endocannabinoids.<br>- - Reinforce tight junction by extracellular vesicles.<br>- Promote intestinal epithelial development by metabolic products
Bacteroides vulgatus	Decreased (q = 0.0408)	Harmful;<br>- *B. vulgatus* protease contributed to UC and transplantation to germfree mice induced colitis.
PAC001081_s group	Decreased (q = 0.0017)	Harmful;<br>- Positively correlated with inflammation in the spleen.

Supplementary Table S4. The abbreviations used in this study

Abbreviation	Full name
DH	Daehong
CP	*Crataegus pinnatifida* Bunge
IBD	inflammatory bowel diseases
DSS	dextran sodium sulfate
5-ASA	5-aminosalicylic acid
DMSO	dissolved in dimethyl sulfoxide
TEER	transepithelial electrical resistance
DCFDA	2',7'-dichlorodihydrofluorescein diacetate
SZ	Smac mimetic and z-VAD-fmk complex
PBS	phosphate buffered saline
ROS	reactive oxygen species
ELISA	enzyme linked immunosorbent assay
H&E	Hematoxylin & eosin
OUT	operational taxonomy unit
ASV	amplicon sequence variants
PCoA	principal coordinate analysis
DAA	differential abundance analysis

Supplementary Figure S1.

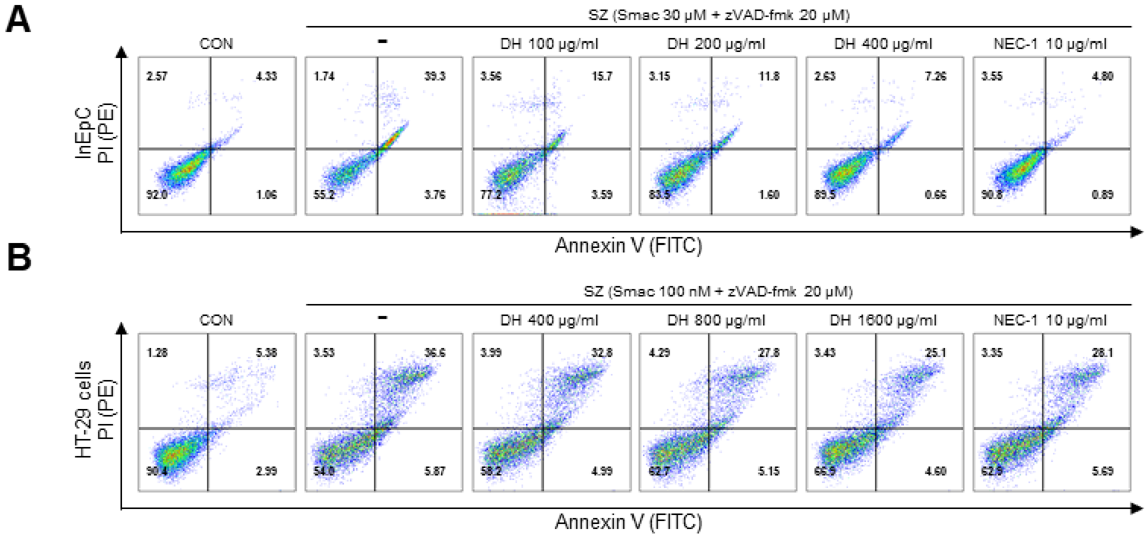

Supplementary Figure S1. DH regulates necroptotic cell death. FACS analysis of annexin V/PI-stained cells in InEpC (A), HT-29 (B).

Supplementary Figure S2.

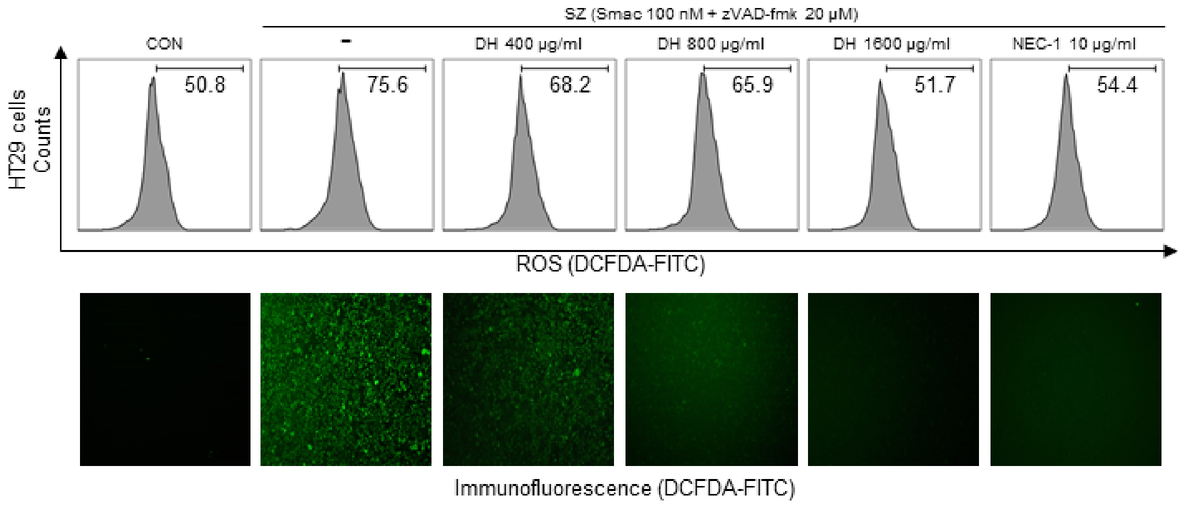

Supplementary Figure S2. DH regulates ROS production. FACS analysis of DCFDA in HT-29 cells.
